# Toward Wide-Field, Extended-Range 3D Vision: A Biomimetic Curved Compound-Eye Imaging System

**DOI:** 10.3390/s26030901

**Published:** 2026-01-29

**Authors:** Songchang Zhang, Xibin Zhang, Yingsong Zhao, Xiangbo Ren, Weixing Yu, Huangrong Xu

**Affiliations:** 1 School of Information Engineering, Xi’an University, Xi’an 710065, China; zhangsc@xawl.edu.cn; 2 Key Laboratory of Spectral Imaging Technology, Xi’an Institute of Optics and Precision Mechanics, Chinese Academy of Sciences, No.17 Xinxi Road, Xi’an 710119, China; zhangxibin@opt.ac.cn (X.Z.); renxiangbo24@mails.ucas.ac.cn (X.R.); yuwx@opt.ac.cn (W.Y.); 3 National Key Laboratory of Solid Rocket Propulsion, Institute of Xi’an Aerospace Solid Propulsion Technology, Xi’an 710025, China; 41_zhaoyingsong@aaspt.casc; 4 Center of Mechanics Science and Optoelectronics Engineering, University of Chinese Academy of Sciences, Beijing 100049, China

**Keywords:** biomimetic optics, compound eye, optical design, wide-field imaging, depth mapping

## Abstract

This work presents a biomimetic curved compound-eye imaging system (BCCEIS) engineered for extended-range depth mapping. The system is designed to emulate an apposition-type compound eye and comprises three key components: a hemispherical array of lenslets forming a curved multi-aperture imaging surface, an optical relay subsystem that transforms the curved focal plane into a flat image plane compatible with a commercial CMOS sensor, and a high-resolution CMOS detector. Comprehensive optical analysis confirms effective aberration correction, with the root-mean-square (RMS) spot radii across the field of view (FOV) remaining smaller than the radius of the Airy disk. The fabricated prototype achieves an angular resolution of 2.5 mrad within an ultra-wide 97.4° FOV. Furthermore, the system demonstrates accurate depth reconstruction within the entire FOV at distances up to approximately 2 m, exhibiting errors below 2%. Owing to its compact form, wide FOV, and robust depth-sensing performance, the BCCEIS shows strong potential as a payload for unmanned aerial vehicles in applications such as security surveillance and obstacle avoidance.

## 1. Introduction

**Figure 1:**
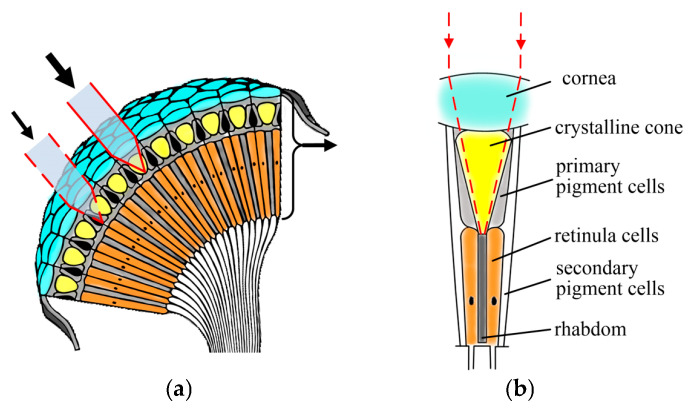
The principle of an apposition compound eye: (**a**) section view of the natural curved compound eye; (**b**) schematic diagram of the cellular organization of the ommatidium.

The compound eye, found in nature, serves as an inspiration for biomimetic optics research owing to its wide field of view (FOV), compact volume, and high sensitivity to moving objects [1,2,3]. As illustrated in Figure 1, the fundamental imaging unit of the compound eye is the ommatidium, which, as shown in Figure 1a, comprises a cornea, a crystalline cone, a rhabdom and pigment cells [4,5]. In apposition-type compound eyes, ommatidia are typically arranged on a curved surface, enabling a wide FOV. Extensive shielding by screening pigment cells between adjacent ommatidia prevents optical crosstalk, thereby ensuring that each ommatidium functions as an isolated imaging channel [6,7,8]. Over the past few decades, significant efforts have been devoted to developing artificial compound eyes that mimic their biological counterparts. In 2001, Tanida et al. introduced a compact imaging system named TOMBO, consisting of a microlens array and photosensitive cells. The system utilized orthogonally oriented polarizers to suppress cross-talk between adjacent units, limiting its application to polarization-sensitive imaging [9]. Moreover, its planar configuration restricted the FOV. Subsequent studies also reported planar compound-eye systems with similarly constrained FOVs [10,11,12,13]. In 2006, Jeong et al. proposed arranging ommatidia on a hemispherical polymer dome to enhance the overall FOV of the artificial compound eye, employing a confocal microscope as an auxiliary readout device [14]. Later, in 2013, Floreano et al. developed CurvACE, a cylindrically curved compound-eye imaging system incorporating a stretchable rectangular array of 42 × 15 microlenses, a flexible interconnection layer, and a photodetector array [15]. When bent into a curved configuration, the system achieved an FOV of 180° × 60° and demonstrated three-dimensional motion detection via optical flow. However, its low resolution restricted it to optical intensity detection. That same year, Ueno et al. presented a compound-eye camera module with a 60° FOV capable of acquiring depth maps, albeit with a limited working distance of approximately 50 cm [16]. In 2019, Zheng et al. proposed an algorithm for reconstructing 3D images by analyzing light position and intensity distribution based on biological compound-eye principles, achieving a standard deviation below 3.1% in 3D positioning over a depth range of 35–325 mm [17]. In 2020, Ma et al. reported a fiber-optic compound eye for target orientation detection, with angular errors of 1.7540° in azimuth and 1.2512° in elevation for an LED at 570 mm [18]. In 2025, Liu et al. reported that a multi-camera array with varying focal lengths was employed to achieve a ranging distance of 100 cm, with a mean absolute error (MAE) of 1.05 [19]. In summary, existing compound-eye systems are generally limited to short working distances (typically around 0.5 m) and offer only moderate accuracy in 3D sensing, despite the practical need for extended-range, high-precision detection [20,21]. However, practical deployment of compound-eye imaging systems in fields such as autonomous navigation, remote surveillance, and robotic perception critically demands the capability to resolve distant targets with both extended working distances and high measurement accuracy—a challenging requirement that remains inadequately addressed by current biomimetic designs. We propose a biomimetic curved compound-eye imaging system (BCCEIS), a multi-aperture system inspired by the arthropod eye. It features a hemispherical array of discrete optical channels to achieve wide-field, motion-sensitive vision with inherent depth-sensing capability. By optimizing the optical relay subsystem, the BCCEIS doubles the angular resolution, overcoming the typical range-accuracy trade-off and enabling high-precision depth mapping with relative errors below <2% at practical distances up to 2.6 m. The system’s performance was further validated at a distance of 4 m. The BCCEIS integrates a fixed-geometry multi-channel imaging array—providing a 97.4° FOV and a 2.5 mrad angular resolution—with an efficient direct depth-retrieval method. This integration ensures sufficient overlapping information for robust depth calculation while achieving computationally streamlined 3D perception. The compact design is suitable for real-world robotic payloads. A 37.5% overlap ratio between adjacent channels further ensures reliable multi-view observation. Collectively, these advances propel biomimetic imaging toward practical deployment in dynamic environments.

## 2. Design of the BCCEIS

The BCCEIS proposed in this work is designed to emulate an apposition-type curved compound eye. Its main components include a lens array, an optical relay subsystem, and a large-scale CMOS image sensor (Imperx, Boca Raton, FL, USA). The lens array, arranged on a hemispherical shell, functions as the curved compound eye and provides a wide FOV. Each individual lens unit operates as an independent optical imaging channel and is therefore referred to as an ommatidium. The array comprises 127 ommatidia in a hexagonal layout. Due to the inherent planar nature of current photoreceptors, an optical relay subsystem is necessary to redirect the incident light and map the curved image onto a planar CMOS focal plane. This subsystem performs three key functions: transforming the image plane, correcting optical aberrations, and preventing optical crosstalk between adjacent channels. Finally, a large-scale CMOS image sensor is employed to ensure the acquisition of high-quality optical images.

### 2.1. Optical Design

**Figure 2:**
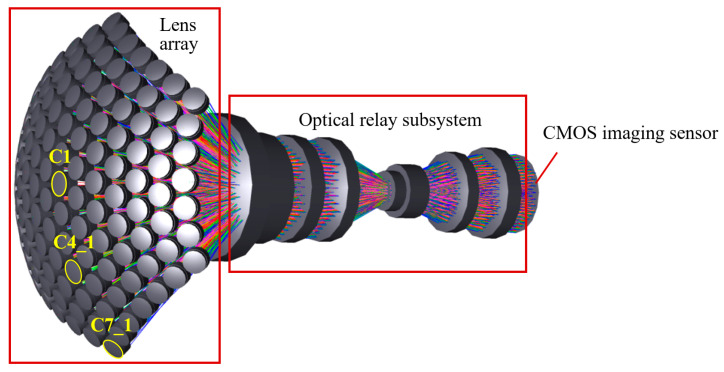
The optical layout for the designed BCCEIS.

Figure 2 illustrates the optical layout of the designed BCCEIS. The system exhibits a focal length of 5 mm and an F-number of 3.5, with a total optical track length of 163 mm. Each ommatidium is implemented as a doublet lens, featuring an aperture of 7.4 mm and a focal length of 17.7 mm. The optical relay subsystem comprises eight lenses, each with a focal length of 14 mm. For optical detection, a CMOS sensor (Imperx, Boca Raton, FL, USA) is employed, offering a resolution of 5120 × 5120 pixels and a pixel size of 4.5 μm × 4.5 μm. To evaluate the imaging performance of the overall BCCEIS, which possesses rotational symmetry, the imaging quality of three representative ommatidia—namely, the central channel (C1), an intermediate channel (C4_1), and the edge channel (C7_1)—was analyzed to characterize the performance of the entire system.

When a point source emits multiple rays through an optical system, diffraction and aberrations prevent the rays from converging to a single point on the image plane. Instead, they form a distribution of scattered points within a certain area, known as a spot diagram. This pattern represents the combined effect of diffraction and geometrical aberrations on the ray positions at various cross-sections near the image plane. The RMS radius of this point distribution provides a measure of its spread and serves as a criterion for evaluating the imaging quality of the optical system. The RMS spot radius is calculated as follows:

(1) $$r_{RMS}=\sqrt{\frac{\sum_{i=1}^{n}r_{i}^{2}}{n}}$$

**Figure 3:**
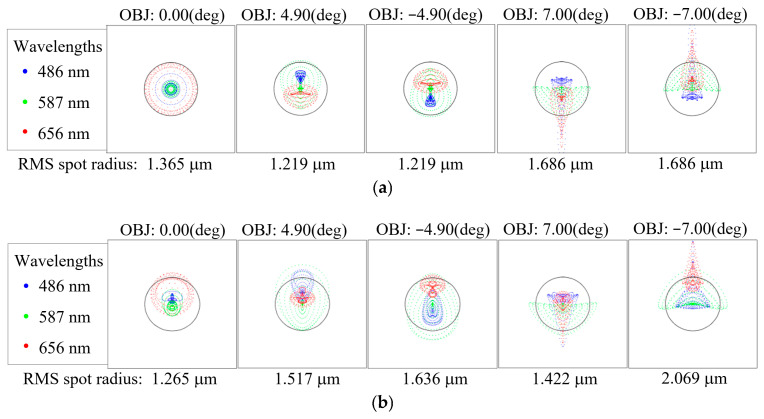

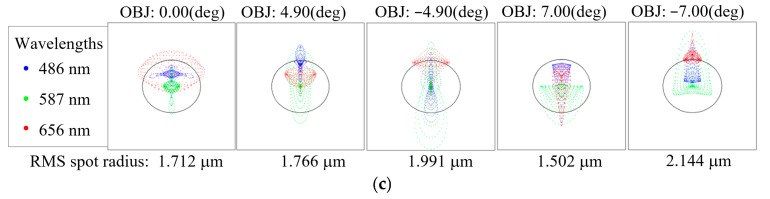
The spot diagrams for Channels 1 (**a**), 4_1 (**b**), 7_1 (**c**).

Here, *r_i_* denotes the radial distance of each point in the spot diagram from the centroid, and *n* represents the total number of sampled points. Figure 3a–c present the spot diagrams for the C1, C4_1, and C7_1 imaging channels of the BCCEIS, respectively. The diagrams illustrate the spot distributions at three wavelengths—486 nm (blue dots), 587 nm (green dots), and 656 nm (red dots)—across the central (0.00°), intermediate (±4.90°), and edge (±7.00°) FOVs for each channel. The ray intersection points on the image plane are represented by scattered dots in the corresponding colors. The black solid circle denotes the Airy disk (RMS radius 2.605 μm) calculated based on the F-number and the wavelength of BCCEIS, representing the diffraction limit of the optical system. As shown, the RMS spot radii for the C1, C4_1, and C7_1 imaging channels range from 1.219 μm to 2.144 μm across all FOVs, all of which are smaller than the Airy disk radius. This indicates that the BCCEIS achieves imaging performance close to the diffraction limit over the full field, demonstrating well-controlled aberrations. Notably, the maximum RMS spot radius (2.144 μm) is smaller than the pixel size (4.5 μm), implying that under infinite-conjugate conditions, the focused spot energy can be effectively collected by a single pixel. This characteristic helps maintain high imaging contrast and signal-to-noise ratio, thereby ensuring the overall imaging performance of the system.

**Figure 4:**
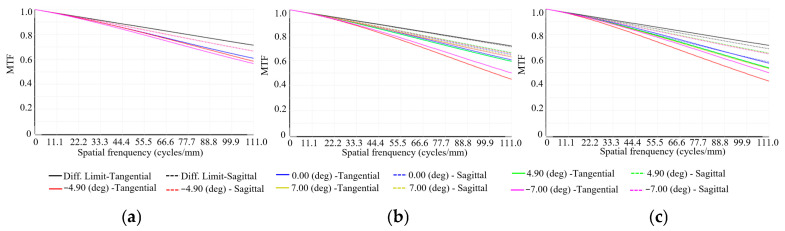
MTF curves for Channels 1 (**a**), 4_1 (**b**) and 7_1 (**c**).

The Modulation Transfer Function (MTF) serves as a quantitative measure of an optical system’s ability to reproduce scene detail, directly characterizing image sharpness. The intensity distribution in an optical image can be expressed as a linear combination of sinusoidal components at various spatial frequencies. The MTF curve, which plots modulation transfer as a function of spatial frequency, describes how faithfully the object’s modulation is transferred to the image through the optical system. It thus provides a key metric for evaluating optical performance. According to the Nyquist sampling theorem, the Nyquist cutoff frequency *N* of the optical system is given by:

(2) $$N=\frac{1}{2p}$$

where *p* represents the pixel size, which is 4.5 μm. Consequently, the Nyquist frequency is calculated as 111 cycles/mm. Figure 4a–c present the polychromatic weighted MTF curves of the three imaging channels (C1, C4_1, C7_1) in the BCCEIS at different FOVs. Polychromatic weighting was applied using wavelengths of 486 nm (25%), 587 nm (50%), and 656 nm (25%). The curves are color-coded by field: blue for the on-axis field (0°), green and red for the positive and negative intermediate fields (±4.90°), and yellow and pink for the positive and negative edge fields (±7.00°), with the black curve representing the theoretical diffraction limit. Solid and dashed lines denote the tangential and sagittal directions, respectively. All curves remain closely grouped and exhibit stable behavior across the spatial frequency range, indicating uniform modulation transfer performance over the full FOV. At the Nyquist cutoff frequency of 111 cycles/mm, the MTF values for C1, C4_1, and C7_1 at the ±7.00° FOVs are 0.57, 0.45, and 0.43, respectively. The MTF values at this cutoff frequency exceed 0.43 from the center to the edge for all channels, demonstrating minimal degradation in spatial resolution and highly consistent imaging quality across the FOV. The close alignment between the tangential and sagittal curves further confirms effective astigmatism correction. Together, these results demonstrate that the BCCEIS achieves nearly diffraction-limited, uniform, and stable optical imaging performance across the entire FOV.

**Figure 5:**
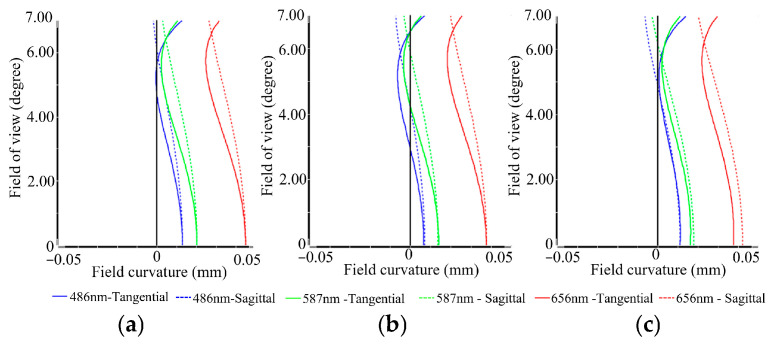
Field curvature for Channels 1 (**a**), 4_1 (**b**) and 7_1 (**c**).

Figure 5a–c illustrate the field curvature distributions across the focal plane for the three imaging channels (C1, C4_1, and C7_1) of the BCCEIS, evaluated at wavelengths of 486 nm, 587 nm, and 656 nm as a function of the FOV. In each plot, the horizontal axis corresponds to field curvature, while the vertical axis denotes field of view. The curves are color-coded by wavelength: blue for 486 nm, green for 587 nm, red for 656 nm, with solid and dashed lines representing the tangential and sagittal directions, respectively. The analysis indicates that all three channels demonstrate well-controlled field curvature characteristics across the evaluated wavelengths. Both tangential and sagittal field curvature curves exhibit smooth profiles and remain closely aligned throughout the entire FOV, with peak field curvature below 0.05 mm. Furthermore, the strong overlap among the curves at the three wavelengths reflects the system’s capability for comprehensive aberration correction, marked by low astigmatism and minimal chromatic variation in field curvature.

These field curvature results are consistent with the earlier MTF and spot diagram analyses, together confirming that the BCCEIS maintains a flat image plane and effectively suppresses aberrations across the full FOV. Such characteristics substantiate the system’s ability to deliver nearly diffraction-limited and highly uniform imaging performance over the entire FOV.

Distortion is defined as the deviation between the ideal image height *y*′ and the actual height *Y_p_*′, where the chief ray intersects the Gaussian image plane. This aberration arises because the lateral magnification β of the optical system varies with the FOV rather than remaining constant. The relative distortion is expressed as follows:

(3) $$dist=\frac{Y_{p}^{\prime }-y^{\prime }}{y^{\prime }}\cdot 100\%$$

**Figure 6:**
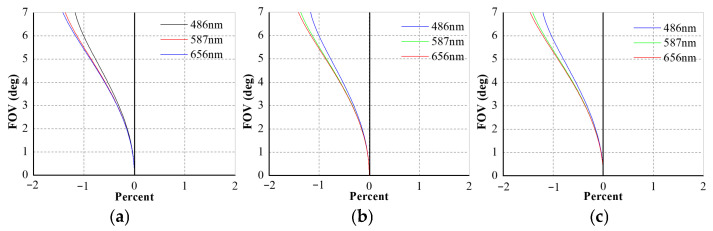
Distortions for Channels 1 (**a**), 4_1 (**b**), and 7_1 (**c**).

Figure 6a–c illustrate the distortion characteristics of the C1, C 4_1, and C7_1 of the BCCEIS, respectively. In the plots, the horizontal axis denotes the relative distortion (in %), the vertical axis corresponds to the FOV, and the line color represents different wavelengths. As can be seen, the distortion values for all channels across the entire FOV remain below 1.5%. Overall, the optical analysis confirms that the designed BCCEIS delivers high-quality imaging performance with well-controlled aberration.

### 2.2. Optical Design Tolerance Analysis

To ensure the manufacturability and alignment feasibility of the designed BCCEIS, a comprehensive tolerance analysis of the optical system was performed. Table 1 summarizes the tolerance budget evaluated at the Nyquist frequency of 111 cycles/mm. The budget is divided into two groups, i.e., the lens array and the optical relay subsystem. The lens array tolerance defines the allowable angular misalignment among the individual lens units, while the relay subsystem tolerance specifies the positional and orientational alignment requirements between the lenses and the mechanical mounts.

**Figure 7:**
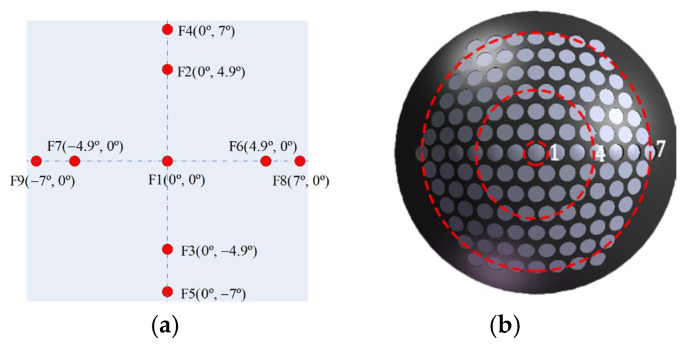
(**a**) Sampled FOVs; (**b**) sampled channels for tolerance analysis (the white circles represent the individual ommatidia. The white circles represent individual ommatidia; the red dashed circles highlight ommatidia in rings 1, 4, and 7.

**Figure 8:**
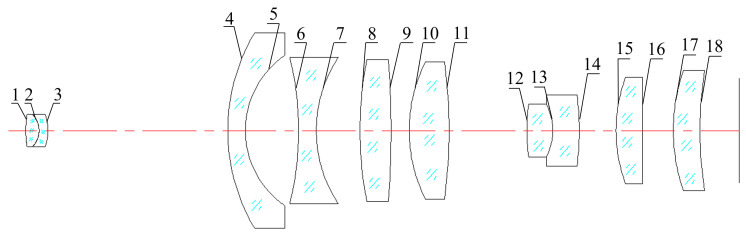
The optical structure diagram for tolerance analysis. The numbers indicate the sequence of the lenses, and the red dash-dotted lines represent the optical axes of the BCCEIS.

To conduct the tolerance analysis, nine representative field points within each channel (Figure 7a) and three regions spanning the full FOV (Figure 7b) were selected. Sensitivity analysis was performed using Zemax’s finite-difference tool. A user-defined merit function was constructed based on the MTF at the diffraction-limited Nyquist frequency of 111 cycles/mm. Here, we clarify the terminology: a sensitivity driver refers to a manufacturing or alignment parameter whose variation most significantly impacts system performance (here, the MTF); in contrast, a tolerance is the allowable range assigned to that parameter. For each tolerance parameter, it was perturbed within its maximum and minimum limits following a standard probability distribution, and the resulting worst-case degradation of the merit function was evaluated for every field point. This approach enables an accurate assessment of the expected performance of the optical system after manufacturing and assembly. The optical layout used for tolerance analysis is shown in Figure 8, where labels 1–18 denote the surface indices of the lenses. Table 2 summarizes the most sensitive tolerances identified through the analysis. As shown, surfaces 1–4, 5, and 15–18 exhibit an MTF degradation of less than 0.005, indicating that the fabrication and assembly tolerances associated with these surfaces have a negligible impact on the image quality of the BCCEIS in practice. For the remaining optical surfaces, the effects on the MTF of surface decenter, surface tilt, element decenter, and element tilt tolerances are listed in Table 2. Overall, the tolerance analysis reveals that, with the exception of surface 13—which requires tighter manufacturing control—no individual tolerance is stringent enough to cause significant degradation in optical performance.

**Figure 9:**
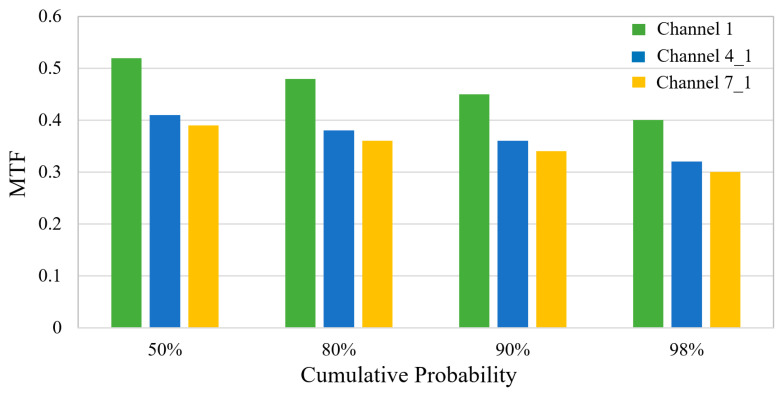
Monte Carlo tolerance analysis results.

The Monte Carlo simulation method, which is well-suited for statistical analysis involving multiple random variables due to its large sample size and high computational accuracy, was employed to further evaluate the tolerance stack-up of the BCCEIS. The simulation consists of two parts: specifying the tolerance ranges (Table 1) and performing statistical analysis. Within the prescribed tolerances, the optical software simulated the resulting MTF variation. A total of 2000 Monte Carlo trials were conducted to predict the as-built performance of a camera prototype under the given tolerance set. The overall statistical results at 111 cycles/mm are presented in Figure 9. The results demonstrate that the allocated tolerances are relatively loose, indicating that the BCCEIS is robust against typical manufacturing and assembly errors. After tolerance stacking, the MTF values at Nyquist frequency decreased to approximately 0.40, 0.32, and 0.30 for Channels 1, 4_1, and 7_1, respectively. The largest probable MTF reduction at a 98% confidence level is −0.08. Across all sampled field points, the MTF remains above 0.35 throughout the entire FOV, confirming that the system maintains acceptable image quality under realistic fabrication and alignment conditions.

### 2.3. Assembly of the BCCEIS

**Figure 10:**
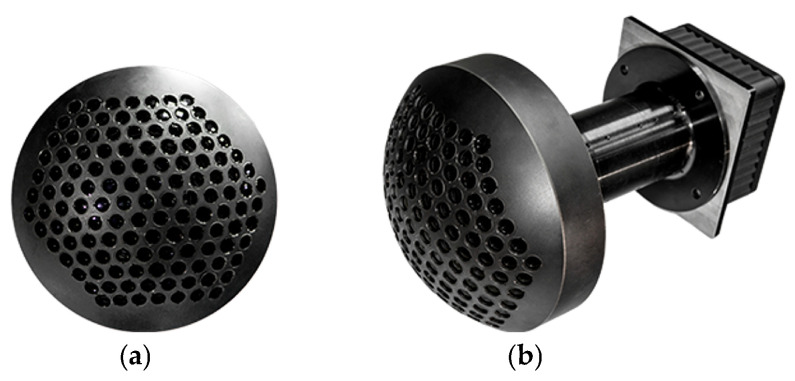
(**a**) The machined hemispherical shell installed with a lens array; (**b**) the integrated prototype BCCEIS.

The primary mechanical component of the system is a metallic hemispherical shell, which serves as the mounting structure for the lens array that forms the compound eye. The shell was fabricated from aluminum alloy using five-axis machining, a process capable of achieving positional accuracy within 5 μm and angular accuracy within 2″. These manufacturing tolerances are well within the limits derived from the earlier tolerance analysis. After machining, the surface of the aluminum alloy shell was anodized to a black finish to suppress stray light. A hexagonal array of through-holes, each featuring a sidewall pedestal for lens seating, was machined into the hemispherical shell to secure the lens array. Each hole has a depth of approximately 8 mm, and the minimum wall thickness between adjacent holes exceeds 1 mm to ensure sufficient structural rigidity. Figure 10 shows the manufactured hemispherical shell with the mounted lens array and fully integrated BCCEIS prototype. The lens array comprises 127 individual lenses arranged hexagonally, enabling an FOV of approximately 97.4°. Key performance parameters of the final prototype are summarized in Table 3.

## 3. Results and Discussion

**Figure 11:**
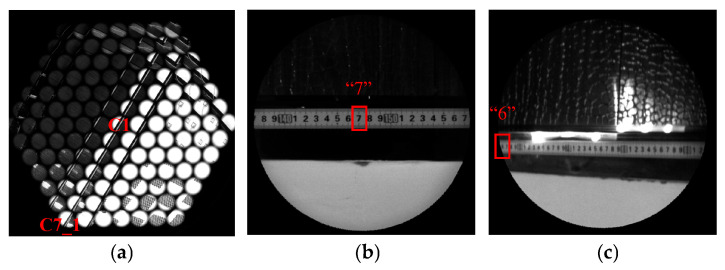

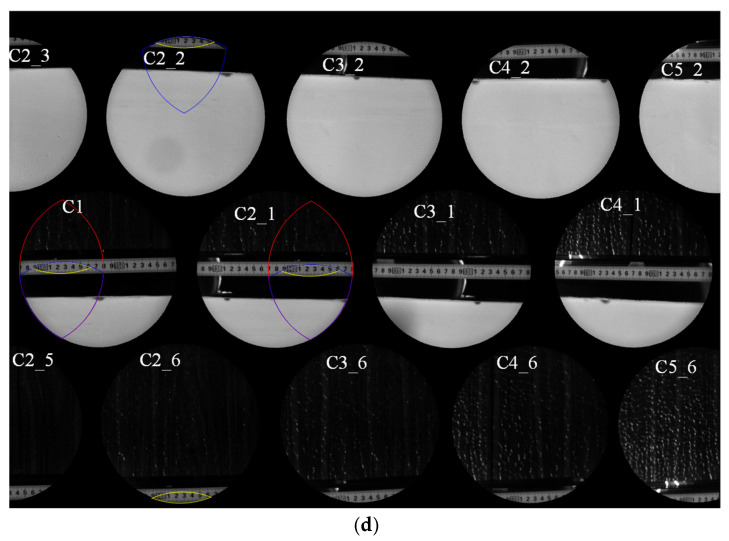
(**a**) Images of the ruler formed by the prototype BCCEIS; (**b**,**c**) Enlarged view of images formed by channels 1 and 7_1 in (**a**); (**d**) Close-up view of (**a**).

Figure 11 presents the imaging results obtained with the developed BCCEIS prototype. A stainless-steel ruler was placed 800 mm in front of the BCCEIS as the target. The center of BCCEIS Channel 1 was aligned directly with the numeral “147” on the ruler. Figure 11a shows the raw image covering the entire FOV of the BCCEIS, which consists of 127 sub-images. Each sub-image has a diameter of 1.22 mm and corresponds to the output of a single ommatidium in the compound-eye array. The visible gaps between adjacent sub-images indicate the absence of optical crosstalk between neighboring ommatidia. Figure 11b and Figure 11c display enlarged views from ommatidia in C1 and C7_1, respectively. All scale markings on the ruler are clearly resolved. As seen in Figure 11b,c, the numeral “7” following “140” appears at the center of the image plane for the C1, while the numeral “6” that follows “50” appears at the edge of the image plane for the C7_1. Therefore, the corresponding length on the ruler from the center to one edge of the FOV is 910 mm. The actual FOV of the BCCEIS can then be calculated using the following formula:

(4) $$\omega =2\times \arctan\left(\frac{910}{800}\right)=97.4{}^{\circ}$$

Given that the finest resolved division on the ruler is 2 mm, the angular resolution of the system is determined to be 2.5 mrad, calculated using the following equation:

(5) $$\theta =arctan\frac{2}{800}=2.5\;mrad$$

It should be noted that Figure 11d clearly demonstrates the presence of overlapping FOVs between adjacent imaging channels. The ruler segment from “139” to “146” is simultaneously and clearly imaged by C1, C2_1, C2_2, and C2_6, while the same segment falls within the imaging blind zone of C2_3 and C2_5. Furthermore, four imaging channels—C2_1, C3_1, C3_2, and C3_6—can simultaneously capture the ruler interval from “129” to “136”, while another set of four channels—C1, C2_1, C2_2, and C2_6—covers the region from “138” to “145”. By performing feature-point matching, the critical boundary of FOV overlap between adjacent sub-images was detected, and the overlap ratios were quantified as follows: the overlap ratio between two adjacent sub-images is 37.5% (marked by the red curve in Figure 11d), that among three adjacent sub-images is 21.8% (blue curve), and that among four adjacent sub-images is 2.2% (yellow curve). In summary, a single object-space target can be captured simultaneously by up to four neighboring ommatidia. This overlapping coverage enables advanced image processing and supports the reconstruction of three-dimensional information. A higher overlap ratio increases the number of ommatidia involved in depth calculation, thereby improving accuracy; however, excessive overlap inevitably constrains the total detectable FOV of the BCCEIS. Therefore, as a trade-off between overlap ratio and FOV coverage, the number of ommatidia forming an overlapping group was optimized to four, resulting in an effective FOV for the BCCEIS of approximately 98°.

**Figure 12:**
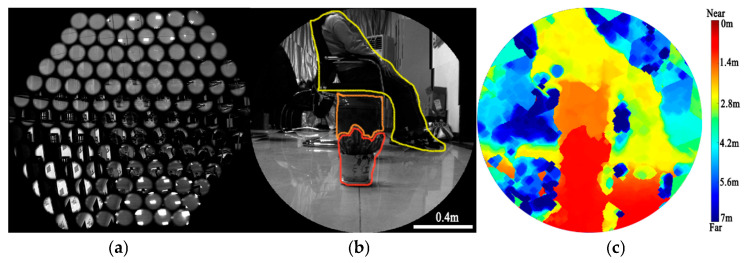
The raw (**a**) and reconstructed (**b**) images formed by the BCCEIS; (**c**) the retrieved.

To evaluate the depth-mapping performance of the system, a scene containing two flowerpots placed 1 m and 1.7 m from the BCCEIS and a woman seated on a bench at 2.6 m was imaged. Figure 12a shows the raw compound-eye image, while Figure 12b presents a reconstructed full-FOV image generated from the raw compound-eye output by exploiting the fixed spatial relationship among individual ommatidia. Using the spatial-to-pixel mapping method, the system converts the multi-channel sub-images into a single, continuous scene suitable for direct human observation.

The BCCEIS employs a multi-ocular stereo-matching algorithm based on Singular Value Decomposition (SVD) in combination with its regularly arranged ommatidia. This approach overcomes the limitations of conventional binocular stereo vision in terms of texture-direction sensitivity and occlusion. The array geometry provides effective baselines in both the horizontal and vertical directions, enabling the system to always select at least one pair of imaging units to generate valid disparity for comprehensive feature matching and depth estimation, regardless of whether the target surface texture is oriented horizontally, vertically, or obliquely. The BCCEIS prototype underwent geometric calibration to establish the precise spatial relationship between adjacent ommatidia. Following established stereo-vision principles [22], multiple regularly arranged imaging units are first used to simultaneously capture multi-view observations of a target. This constructs a system of linear equations that incorporates the projection relations from all views. Next, the scale factor is algebraically eliminated, which formulates a global optimization problem for the 3D coordinates of the target point. The coefficient matrix is then subjected to singular value decomposition, and the right singular vector corresponding to the smallest singular value is extracted as the optimal spatial coordinates, thereby minimizing the total projection error across all ommatidia in the least-squares sense. This method effectively integrates multi-view geometric constraints, achieves stable matching for textures in any orientation by virtue of the dual-baseline configuration, and naturally suppresses the effects of occlusion and noise through a global optimization mechanism, ultimately enabling high-accuracy and high-robustness depth reconstruction. Using the SVD-based multi-ocular algorithm to compute depth values, the resulting depth map is shown in Figure 12c. The measured depths for the three targets (the two flowerpots and the woman) are 0.993 m, 1.67 m, and 2.56 m, respectively. The relative errors are all within 2% of the ground-truth values, confirming the system’s precise depth-mapping capability within the evaluated range. Additionally, during depth estimation, the search range was constrained to 1–4 m. The measurement error correlates positively with distance, yielding a 5% error at 3.2 m and a 6.2% error at 4 m.

## 4. Conclusions

This study presents the design, fabrication, and experimental validation of a BCCEIS capable of high-resolution imaging and depth mapping at extended working distances. The system comprises 127 ommatidia arranged on a hemispherical shell, achieving an ultra-wide FOV of 97.4°. Optical simulations and laboratory measurements confirm an angle resolution of 2.5 mrad and an MTF exceeding 0.4 at the Nyquist frequency of 111 cycles/mm, demonstrating imaging quality suitable for distances beyond 1 m. The design employs conventional optical materials and standard surface geometries, facilitating manufacturability and alignment within loose tolerance budgets, as verified by sensitivity and Monte Carlo analyses. By exploiting the intentional overlap between adjacent ommatidia, full-FOV image reconstruction and depth estimation were implemented using a calibrated stereo-vision pipeline combined with semi-global block matching. Experimental results on targets placed between 1 m and 2.6 m show depth errors below 2%, confirming the system’s accuracy in 3D sensing. In summary, the BCCEIS provides a practical and scalable approach to achieving a wide FOV, high angular resolution, and robust mechanical design, making it suitable for applications requiring simultaneous high-resolution 2D imaging and 3D mapping, such as autonomous navigation, surveillance, and robotic vision.

## Figures and Tables

**Table 1:** Tolerance budget for BCCEIS.

Tolerance Type	Lens	Optical Relay Subsystem
Surface decenter (TSDX, TSDY)	±25 μm	±20 μm
Surface tilt (TSTX, TSTY)	±1 mrad	±0.35 mrad
Element decenter (TEDX, TEDY)	±25 μm	±20 μm
Element tilt (TETX, TETY)	±2 mrad	±0.35 mrad
Thickness (TTHI)	±50 μm	±50 μm

**Table 2:** Key sensitivity drivers and their allocated tolerance budget.

Tolerance Type	Tolerance Value	Affected Surface	Worst MTF Value Drop (@111 Cycles/mm)
TSDY	±20 μm	13	−0.018
TEDY	±20 μm	10–11	−0.015
TSDX	±20 μm	13	−0.012
TSDY	±20 μm	7	−0.011
TSDY	±20 μm	10	−0.010
TEDY	±20 μm	6–7	−0.009
TSDY	±20 μm	13	−0.008
TSDY	±20 μm	5	−0.007
TEDY	±20 μm	4–5	−0.006
TSTX	±0.35 mrad	8	−0.006
TEDX	±20 μm	12–14	−0.005

**Table 3:** The performance parameters of the BCCEIS.

Parameter	Value
Working waveband	Visible
f-number F/#	3.5
Number of Ommatidia N	127
Total FOV ω	97.4°
Acceptance angle of each ommatidium Δφ	14°
Inter-optical-axis angle ΔΦ	7°
Radius of the hemispherical shell R	68 mm
Diameter of lens in lens array D	7.4 mm
Angle Resolution r	2.5 mrad
MTF at 111 cycles/mm	>0.4
Distortion per channel	<1.5%
Overall dimensions	Φ123 mm × 195 mm (Diameter × Length)
Whole weight W	1.2 kg
Power P	6.5 W

## Data Availability

The original data presented in this study are openly available in Zenodo at https://doi.org/10.5281/zenodo.18058325.
